# Differential CMOS Sub-Terahertz Detector with Subthreshold Amplifier

**DOI:** 10.3390/s17092069

**Published:** 2017-09-09

**Authors:** Jong-Ryul Yang, Seong-Tae Han, Donghyun Baek

**Affiliations:** 1 Department of Electronic Engineering, Yeungnam University, Gyeongsan, Gyeongbuk-do 38541, Korea; jryang@yu.ac.kr; 2 Electric Propulsion Research Center, Korea Electrotechnology Research Institute, Changwon, Gyeongnam-do 51543, Korea; saiph@keri.re.kr; 3 School of Electrical Engineering, Chung-Ang University, Seoul 06974, Korea

**Keywords:** CMOS integrated circuit, differential detector, integrated antenna, raster scanning, subthreshold amplifiers, THz detector, THz imaging

## Abstract

We propose a differential-type complementary metal-oxide-semiconductor (CMOS) sub-terahertz (THz) detector with a subthreshold preamplifier. The proposed detector improves the voltage responsivity and effective signal-to-noise ratio (SNR) using the subthreshold preamplifier, which is located between the differential detector device and main amplifier. The overall noise of the detector for the THz imaging system is reduced by the preamplifier because it diminishes the noise contribution of the main amplifier. The subthreshold preamplifier is self-biased by the output DC voltage of the detector core and has a dummy structure that cancels the DC offsets generated by the preamplifier itself. The 200 GHz detector fabricated using 0.25 μm CMOS technology includes a low drop-out regulator, current reference blocks, and an integrated antenna. A voltage responsivity of 2020 kV/W and noise equivalent power of 76 pW/√Hz are achieved using the detector at a gate bias of 0.5 V, respectively. The effective SNR at a 103 Hz chopping frequency is 70.9 dB with a 0.7 W/m^2^ input signal power density. The dynamic range of the raster-scanned THz image is 44.59 dB.

## 1. Introduction

The plasmon detection using complementary metal-oxide-semiconductor (CMOS) field-effect transistors is a promising technology for a terahertz (THz) imaging system with focal plane arrays. The CMOS plasmon detector has many advantages such as room temperature operation, fast response time, and easy integration with read-out and control circuits [1,2,3,4]. High responsivity and low noise characteristics are the main issues in applying the CMOS plasmon detector to an imaging system for security screening and non-destructive testing of materials [2,3,4].

Highly sensitive detectors that employ a differential antenna and integrated amplifiers were developed in the previous studies [5,6,7,8,9,10,11]. These detectors were directly connected to high gain amplifiers in the back of the detector core [5,8,9,10,11]. The detectors with a differential antenna can avoid the parasitic antenna effect [12]. However, the noise generated in the amplifiers and control circuits for pixel selection in the array lowers the signal-to-noise ratio (SNR) of the detector. Adding an amplifier for every pixel detector in the array increases power consumption and chip area and it is also difficult for them to efficiently extract the signals from the differential antenna in the signal conditioning block (SCB), which amplifies them and filters out the noises. Device mismatches and DC offsets in the block can decrease the efficiency of the signal summation [13]. In addition, the overall noise level of the detector increases because the noise generated in the SCB is added to that of the overall detector. In addition, the detector becomes sensitive to interference from the interconnection lines between the detector and SCB [12,14]. Therefore, improving the SNR for a THz imaging system is difficult, even though the responsivity was shown to increase in the detectors reviewed in previous studies.

In this study, a CMOS integrated circuit (IC) for the plasmon detector using a subthreshold preamplifier is proposed to improve the responsivity and effective SNR for THz imaging systems. The differential outputs of the detector core are amplified at the subthreshold region with low-power consumption and are combined. The noise contribution of the main amplifier to the total noise of the detector is reduced by the gain of the preamplifier. Given that the preamplifier operates only when the sub-THz signals are input to the detector, the effective SNR at the output of the main amplifier can increase. A detector operating at 200 GHz is realized with a differential on-chip antenna and bias circuits using a 0.25 μm CMOS process. Section 2 describes the operating principle and architecture of the proposed detector. The design and implementation of the detector, including the differential patch antenna, bias circuits, and amplifiers, are presented in Section 3. Section 4 provides the measured characteristics of the detector and describes the experimental setup. A conclusion is presented in Section 5.

## 2. Operating Principle and Architecture of the Proposed CMOS Detector

**Figure 1:**
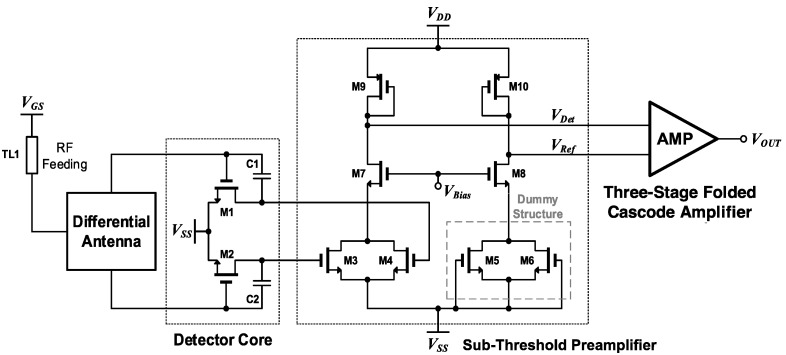
Architecture of the proposed CMOS sub-THz detector.

**Figure 2:**
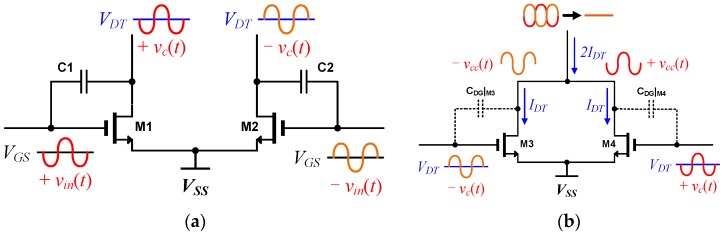
Operating principles of (**a**) the detector core; and (**b**) the transconductance stage of the subthreshold preamplifier in the proposed CMOS sub-THz detector.

The architecture of the proposed sub-THz CMOS detector is shown in Figure 1. The proposed detector consists of a detector core, a subthreshold preamplifier with a cascode configuration, and a folded cascode main amplifier. The main amplifier has a high voltage gain. The THz signals through the differential patch antenna integrated on the chip are transmitted to the M1 and M2 gate nodes in the detector core. The THz signals are also connected to the drain nodes of M1 and M2 through the coupling capacitance C1 and C2 and the DC output voltages *V_DT_* as shown in Figure 2a are generated at the drain node by self-mixing of these signals at M1 and M2 [1,2,5,15]. The DC outputs *V_DT_* of the detector core and THz signals *v_c_*(*t*) coupled through C1 and C2 are applied to the gate nodes of M3 and M4 in the preamplifier. Since *V_DT_* is a function of the gate bias of M3 and M4, the drain DC currents *I_DT_* of M3 and M4 are also determined by *V_DT_* of the detector and the transconductance of M3 and M4. These differential currents are merged at the drain nodes of M3 and M4 in the preamplifier. Since the odd harmonics included in the outputs of the detector core are cancelled out at the drain, the noise from the odd harmonics are eliminated also. M3 and M4 are biased at the subthreshold region due to the low DC outputs of the detector core, which are determined by the intensity of the input THz signals. The DC output voltages that are generated through self-mixing at M3 and M4 are negligible because the coupled differential signals *v_cc_*(*t*) are cancelled out at the drain nodes of M3 and M4. The detection signal is converted to a voltage *V_Det_* in the p-channel metal-oxide-semiconductor (PMOS) load M9 and transmitted to the input voltage of the main amplifier. The dummy structure using M5 and M6 in Figure 1 has the same circuit configuration with the transconductance stage using M3 and M4 except the zero gate voltages. The structure cancels the DC offsets generated in the preamplifier itself, which can distort the output of the detector.

The preamplifiers turn on only when input sub-THz signals are coupled to the antenna and the DC outputs are generated at the output of the detector core. The output voltage difference of the differential antenna is effectively increased by high transconductance of the preamplifier operating at subthreshold region. The effective SNR of the proposed detector substantially affects the operation of the THz imaging application. The effective SNR indicates the voltage difference of the detector output depending on whether there is a sub-THz signal. The effective SNR of the proposed detector can be improved by the self-biased operation of the preamplifiers and by harmonic noise cancellation. When the preamplifier is self-biased by the THz input signals, the noise of the preamplifier is added to the overall noise of the proposed detector. However, the overall noise of the detector decreases due to the preamplifier because the effect of the noise generated in the main amplifier decreases with the gain of the preamplifier. The overall noise of the proposed detector does not significantly increase compared to that of the other detectors described in previous studies. This is because the preamplifier operating at the subthreshold region has less noise than the main amplifier operating at the saturation region [16].

The voltage responsivity *R_V_* and noise figure *N_F_* of the proposed detector can be expressed as

(1) $$R_{V}=2R_{VD}\cdot {A_{V}|}_{Sth}\cdot {A_{V}|}_{P},$$

(2) $$N_{F}=2N_{FD}+{N_{F}|}_{Sth}+\frac{{N_{F}|}_{P}}{{A_{V}|}_{Sth}}$$

where *R_VD_* is the voltage responsivity in each transistor of the detector core, *A_V_|_Sth_* is the voltage gain in the subthreshold preamplifier, *A_V_|_P_* is the voltage gain in the amplifier, *N_FD_* is the noise figure of the detector core, *N_F_|_Sth_* is the noise figure of the preamplifier, and *N_F_|_P_* is the noise figure of the amplifier [17].

## 3. Design and Implementation of the Proposed Detector

### 3.1. Detector Core and Subthreshold Preamplifier

The frequency response of the detector depends on the transistor physical size of the detector core because the intrinsic parasitic capacitance limits the operating frequency [18]. In the proposed detector, the minimum-sized transistors are used in the semiconductor fabrication to enhance the response at 200 GHz. The additional 0.2 pF capacitors are connected between the drains and gates of the transistors. The gate bias of the transistors is connected at the virtual ground node of the integrated antenna to avoid the parasitic effects caused by signal pads, bonding wires and interconnection lines.

**Figure 3:**
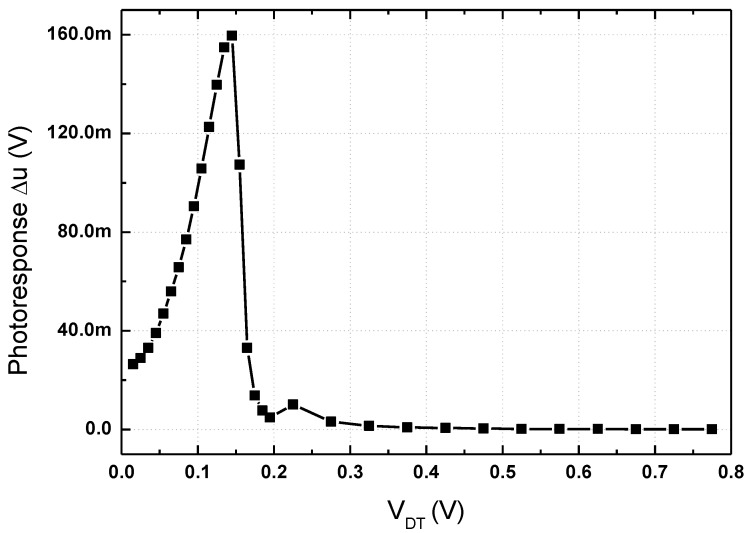
Simulated photoresponse at the output of the subthreshold preamplifier depending on the DC output voltage of the detector core.

The sizes of the transistors in the subthreshold preamplifier are determined by the transconductance and threshold voltage level. If the DC output voltage of the detector core increases over the threshold voltage of transistors M3 and M4 because of increase in the intensity of the THz input signal, the outputs of the preamplifier could be saturated by the dynamic range, and the overall voltage responsivity would be decreased by the operating point. The simulation results show that the output voltage of the detector core is under the threshold voltage of the transconductance stage in the preamplifier. The simulation shown in Figure 3 indicates that when the THz signal power coupled on the integrated antenna is less than −20 dBm, the preamplifier is designed to have a high photoresponse and wide dynamic range at the output of the preamplifier. The width and length of each transistor of the detector core and preamplifier are shown in Table 1.

### 3.2. Three-Stage Folded Cascode Amplifier

**Figure 4:**
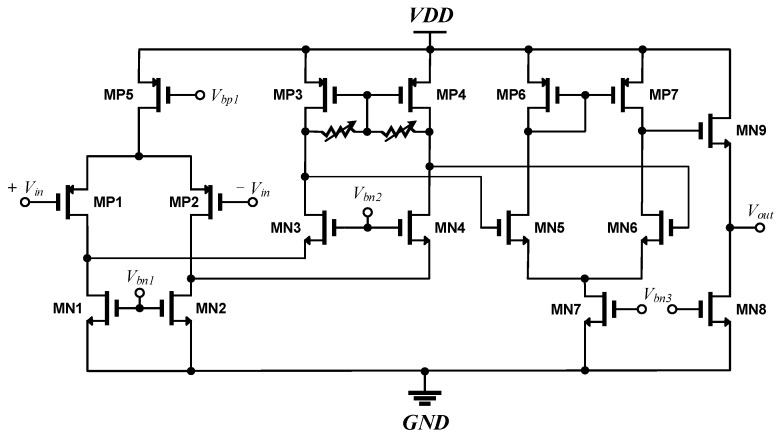
Schematic of the three-stage folded cascode amplifier.

The three-stage folded cascode main amplifier shown in Figure 4 is integrated on a chip. The main amplifier amplifies the voltage difference between the output voltage *V_Det_* of the preamplifier and the reference voltage *V_Ref_* from the dummy structure. The PMOS pair is used in the input stage to reduce the input-referred flicker noise and offset [8]. A folded cascode topology is used in the first stage of the main amplifier for a wide-input common-mode range, high open-loop gain, and wide 3 dB frequency bandwidth [8,19,20]. The frequency bandwidth of the main amplifier is designed to be higher than the modulation frequency. The common-source differential amplifier in the second stage provides a single-ended output conversion. The source follower circuit in the last stage is used as a voltage buffer to obtain low output impedance. All bias voltages of the amplifier in Figure 4 are generated by a proportional-to-absolute temperature current from the bias circuit.

The voltage gain of the main amplifier is from 36 to 44 dB in eight steps. In addition, the 3 dB bandwidth of the main amplifier is greater than 3.9 MHz at all process, voltage and temperature (PVT) corners in simulation. The overall current consumption of the main amplifier during the simulation is 0.9 mA at 1.8 V, which is generated from a 2.5 V supply voltage using a low drop-out (LDO) regulator.

### 3.3. Differential Patch Antenna

**Figure 5:**
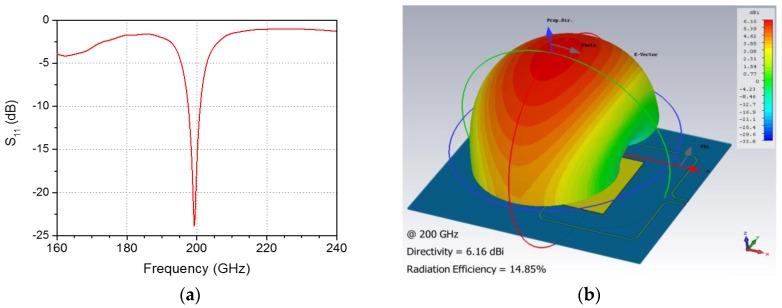
Simulation results of the integrated patch antenna: (**a**) S_11_ and (**b**) the far-field radiation pattern at 0.2 THz.

The differential antenna integrated with the detector is designed for efficient coupling of the THz signals using three-dimensional electromagnetic simulation to operate at 200 GHz. The differential antenna is useful in THz detectors in an imaging system compared to the single-ended antenna, which is susceptible to the effects of the parasitic antenna attributed to the interconnection lines. The input impedance of the linearly polarized patch antenna is designed to be 50 Ω at the differential port. A top-metal aluminum layer that is 3 μm thick is used as a patch layer. The height from the top layer to the patterned ground in a metal-1 layer is 5.71 μm. THz signals via the antenna, the size of which is 465 μm × 353.8 μm, are differentially transmitted to the detector through probe feeds and interconnection lines that are 8.8 μm wide and 0.57 μm thick. In our simulation of the operating frequency, an antenna bandwidth of 5 GHz, radiation efficiency of 14.85%, and directivity of 6.16 dBi were obtained, as shown in Figure 5.

### 3.4. Implementation of the Proposed Detector Using CMOS Process

**Figure 6:**
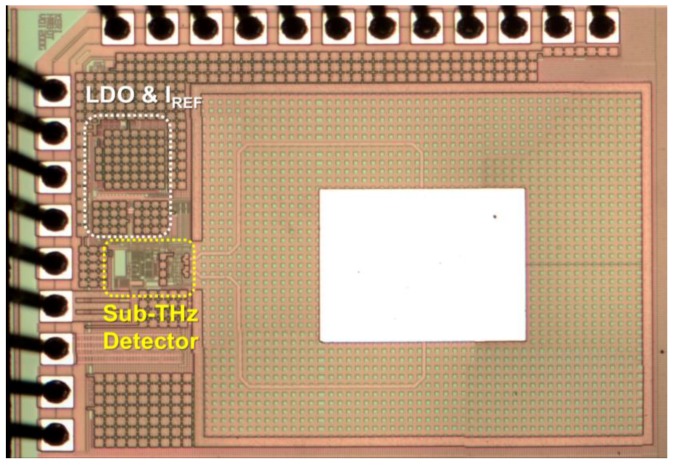
Die photograph of the differential CMOS sub-THz detectors (active dimensions: 390 μm × 220 μm without the antenna area).

The proposed detector was fabricated using a TSMC (Hsinchu, Taiwan) 0.25 μm mixed-signal CMOS process with one poly and five metal layers, as shown in Figure 6. The total area of the detector, including integrated antenna, LDO regulator, and current reference circuit, was 1.4 mm × 1.0 mm, and the active dimensions without the 1 mm × 0.8 mm antenna area were 390 μm × 220 μm. Metal layers that were 20 μm wide, which were connected from a metal-1 to a top-metal layer, were used for device isolation between the antenna and circuits.

## 4. Measurement Results and Discussion

### 4.1. Performance of the Proposed Detector IC

**Figure 7:**
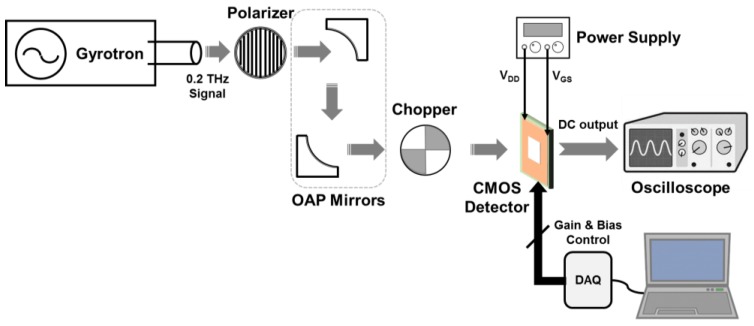
Measurement setup for calculating the voltage responsivity of the proposed detector.

Figure 7 shows the measurement setup for calculating the voltage responsivity of the proposed detector. The 200 GHz signal generated by a gyrotron [21] was guided by the off-axis parabolic (OAP) mirrors and sent to the detector after passing through a polarizer and chopper. The polarizer was used to vary the intensity of the signal to the antenna. The power density of the input signal was calculated to be 0.7 W/m^2^ on these chips [21]. The receiving area was assumed to 1.27 mm × 0.87 mm from the length between the grounded metal lines around the integrated antenna, and the effective antenna area was calculated to 6.98 × 10^−7^ m^2^ from the receiving area, sub-THz frequency, and antenna gain [9].

**Figure 8:**
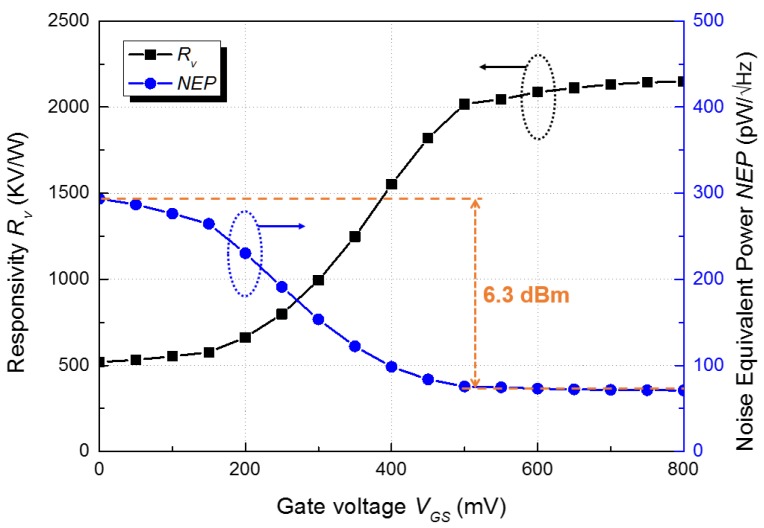
Measured responsivity and noise equivalent power of the proposed detector at 200 GHz.

The response of the detector was monitored by an oscilloscope, which measured the output voltage difference of the detector core. The voltage responsivity as shown in Figure 8 was obtained using the measured output difference and effective electric area for the integrated antenna [5]. In all the measurements presented in this study, the voltage gain of the main amplifier was set to 36 dB. The voltage responsivity of the proposed detector was increased as the gate bias of the detector core increased and was saturated when the gate bias was higher than 0.5 V. This result implies that the gate bias of the detector core affects the gain of the subthreshold preamplifier because the outputs of the detector core are the bias voltages of the preamplifier in the proposed detector. The voltage gain of the main amplifier is the dominant factor of the responsivity in the proposed detector compared to the photoresponse at the output of the preamplifier shown in Figure 3. The output characteristics of the detector core and the preamplifier have an optimum point depending on the gate-source voltage of the detector core (*V_GS_*), but the voltage gain of the amplifier increases as *V_GS_* increases because *V_GS_* affects the bias point of the transistor in the first stage of the folded cascode amplifier. The responsivity saturation as shown in Figure 8 was caused by the voltage saturation at the outputs of the preamplifier and main amplifier when the gate bias of the detector core was higher than 0.5 V.

**Figure 9:**
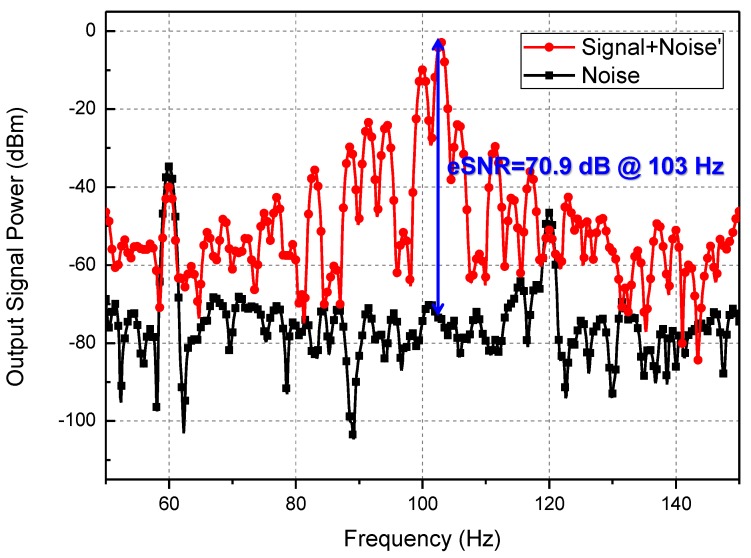
Effective signal-to-noise ratio (SNR) at the output of the proposed detector using the 103 Hz chopping frequency and spectrum analyzer.

Figure 8 also shows that noise equivalent power (NEP), which was measured using a spectrum analyzer instead of the oscilloscope (Figure 7), decreases as the gate bias increases because the noise contribution of the amplifier is reduced by the gain of the subthreshold preamplifier. The NEP is generally used to present the noise characteristics of the plasmon detector because of the inherent detection mechanism of the detector. The output power spectra in Figure 9 show the effective SNR of 70.9 dB at a 103 Hz chopping frequency. The SNR at 103 Hz is approximately 64.6 dB when the noise floor is increased to the maximum value of 6.3 dBm, as shown in Figure 8, by the operation of the preamplifier. It is difficult to obtain the corner frequency of 1/f noise and the input-referred voltage and current noise amplitudes of the amplifiers due to the self-biased configuration of the amplifiers and high operating frequency of the detector. The crucial characteristic of the proposed detector for a THz imaging system is the effective SNR (rather than the standard SNR), which is shown in Figure 9, because the dynamic range of the THz image is practically determined by the output signal difference between the pixel fully covered by the object and the pixel in which the signal is fully coupled with the integrated antenna. The high effective SNR of the proposed detector is one of the figure of merits in realizing a THz imaging system with high image quality and wide dynamic range. In addition, it implies that the effective SNR can be attributed to the high voltage responsivity, the minimized noise level of the detector in operation, and the high input signal power generated by the gyrotron. The multiple peaks in Figure 9 are caused by the low frequency stability and vibration of the mechanical chopper. They are neglected in the calculation of the effective SNR because the similar peaks are found in the noise spectrum. The results show that the responsivity of the detector improved considerably compared with the previous results (Table 2).

### 4.2. THz Imaging with the Proposed Detector Using Raster Scan Method

**Figure 10:**
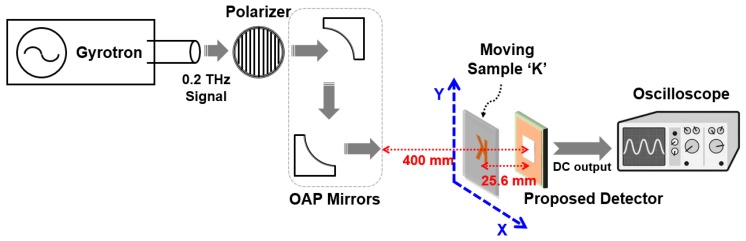
Measurement setup of THz imaging using the detector.

**Figure 11:**
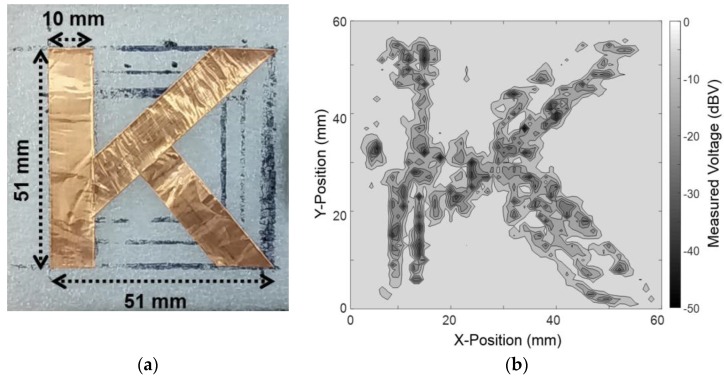
(**a**) A photograph of the moving sample with a ‘K’ shape and size of 51 mm × 51 mm; (**b**) measured THz image for the sample.

The THz imaging using the proposed detector is demonstrated in Figure 10. The THz image of a single target sample was obtained by the raster scan method [23]. The sample was positioned between the OAP mirrors and detector, and the distance from the detector to the sample was 25.6 mm. The output of the detector was measured in each position when the sample moved horizontally or vertically with a step of 1 mm. The step size was determined by considering the area of the integrated antenna. The sample with a ‘K’ shape, whose line width was 10 mm, was patterned by 0.06 mm-thick copper tape and attached to the polystyrene board with a dielectric constant of 1.03 and a thickness of 10 mm. The dimensions of the pattern were 51 mm × 51 mm, as shown in Figure 11a.

The “K” pattern was successfully reconstructed by the imaging system using the proposed detector in Figure 11b. The voltage ratio between the maximum and the minimum outputs in the measurement results for THz imaging was 44.59 dB, which was the dynamic range of the THz image. Assuming that the flicker noise was the dominant noise under 100 Hz in the proposed detector, we concluded that the ratio was nearly similar to the SNR of the detector that was calculated from the effective SNR measured at 103 Hz and noise level increased by the subthreshold amplifier. The poor quality of the THz image in Figure 11b is caused by the low linearity of the proposed detector despite high effective SNR of the detector. Since the linearity of the detector shows how the THz image can be expressed in a constant ratio within the dynamic range, the image quality in the dynamic range is more influenced by the linearity. The nonlinearity is prominent in the proposed detector because of nonlinear characteristics of the plasmon detector and the self-biasing operation in the amplifiers. These show that the performance of the detector, especially the SNR and linearity, determines the quality of the THz image.

The image resolution of less than 10 mm was verified because the 10 mm line width in the measured image could be clearly discriminated, as shown in Figure 11. If the measured line width was defined by the boundary in which the ratio of the output voltage between two adjacent pixels was greater than 5 dB, then the line width of the THz images ranged from the minimum value of 5 mm to the maximum value of 12 mm. The incident THz waves were diffracted and scattered by the target sample, especially at the edge of the pattern. The diffracted waves were transmitted to the detector because the 10.6 mm-thick metal socket, used to fix the detector on the measurement board, functioned as the waveguide for the THz waves. The THz signal coupled to the integrated antenna of the proposed detector was the sum of directly incident waves and diffracted waves, which resulted in some errors at the edges of the pattern.

## 5. Conclusions

A CMOS sub-THz detector with high responsivity employing a self-biased integrated preamplifier is proposed. The subthreshold preamplifier in the proposed detector effectively improves the responsivity by amplifying and combining the outputs of the differential detector core. The SNR is also increased because the preamplifier operates only when the input signals is received in the detector and it has lower noise characteristics than the main amplifier. The detector operating at 200 GHz shows a 2020 kV/W *R_V_*, 76 pW/√Hz NEP, and 70.9 dB effective SNR at the gate bias of 0.5 V and 103 Hz chopping frequency, respectively. The THz imaging is demonstrated with the proposed detector using the raster scan method.

## Figures and Tables

**Table 1:** The width and length of the transistors in the detector core and the preamplifier.

Transistors	Width [μm]	Length [μm]	Operation
M1 & M2	0.3	0.24	Detector core
M3 & M4	1.0	0.35	Transconductance stage
M5 & M6	1.0	0.35	Dummy structure
M7 & M8	18.0	1.0	Additional gain and isolation
M9 & M10	1.0	12.0	Active load

**Table 2:** Comparisons of the sub-THz detectors.

Ref.	Freq. [GHz]	CMOS Technology	Responsivity ^1^ [kV/W]	NEP [pW/√Hz]
[1]	292	0.13 μm	5	8
[2]	650	0.25 μm	80	300
[8]	280	0.13 μm	250	33
[9]	856	65 nm	140	100
[10]	365	90 nm	1200	200
[11]	270	0.13 μm	300	18.7
[22]	290	0.18 μm	0.7	261
This work	200	0.25 μm	2020 ^2^	76

^1^ The voltage gain of the amplifier was different in each detector. ^2^ The measurement result at the gate bias of 0.5 V.
